# Translation and validation of urdu version short form-mcgill pain questionnaire-2

**DOI:** 10.1186/s13102-023-00715-2

**Published:** 2023-08-14

**Authors:** Amna Sharif, Fareeha Amjad, Syed Asadullah Arslan, Ashfaq Ahmad

**Affiliations:** 1 University Institute of Physical Therapy, University of Lahore, Lahore, Pakistan; 2 Riphah International University, Lahore, Pakistan

**Keywords:** Patient reported outcome measure, Validity, Reliability, Chronic pain, Urdu version, And translation

## Abstract

**Background:**

Low back pain is one of the most common complaints affecting many individuals. The McGill Pain Questionnaire is used in various clinical settings to assess different types of pain and one of the most extensively used outcomes measures for pain in the world. The purpose of this study was to translate and validate the original English version of the SF-MPQ-2 into Urdu (SF-MPQ-2-U).

**Methods:**

For this study, Mapi Research Trust protocols were followed for the forward and backward translation. Test-retest reliability was used to assess the reliability. Cronbach’s alpha and Omega was used to determine internal consistency. Pearson’s correlation was used to evaluate convergent validity. Confirmatory factor analysis was also conducted.

**Results:**

The Cronbach’s alpha for SF-MPQ-2-U was 0.73 to 0.79, indicating acceptable internal consistency. Omega score for the SF-MPQ-U were 0.918. The ICC varied from 0.799 to 0.878 for domains of SF-MPQ-2-U. The CFA of the SF-MPQ-2-U met model fit indices with GFI and NFI > 0.90. The inter-scale correlation between baseline and re-test data was from 0.63 to 0.71, indicating a positive and strong correlation. The SF-MPQ-2-U and ODI-U had a baseline correlation of 0.547. The correlation of SF-MPQ-2-U & VAS at baseline data was 0.558. Pearson’s correlation between subscales was r = 0.253 with p 0.01, which was statistically significant.

**Conclusion:**

The SF-MPQ-2-U is considered to have good convergent validity at inter scale and between two scale levels. Reliability was checked by test-retest reliability, Internal consistency was checked using Cronbach’s alpha and Omega that showed good internal consistency for measuring different types of pain in patients with low back pain who speak Urdu. To make the questionnaire more valid and reliable, it is recommended for the researchers to do in-depth research on larger sample size.

## Background

Pain is one of the most prevalent sensations of discomfort among every age group, regardless of male or female, and can cause sleep issues and psychological distress in the patient. Chronic pain is one of the reasons that causes people to get medical care linked to functional limitations [1]. Pain can be described as “An unpleasant sensory and emotional experience associated with, or resembling that associated with, actual or potential tissue damage” [2]. Pain is a complicated physiological and psychological experience, which is difficult to analyze and cure because pain is a subjective sensation. Individual differences in pain perception make assessing and managing pain more difficult. Regardless of whether pain is measured in a clinical or research setting, subjective reporting is one of the most commonly used methods for determining the presence of pain [3]. Pain always has a psychological element because sometimes it is a psychological experience that involves the ideas of suffering and harm and is totally unrelated to actual physical damage [4]. How the patient feels about their pain depends on cultural and environmental factors. These factors lead to a difference in how the patient deals with the problem and gets treatment. Cultural and environmental factors also influence the reporting style of the patient about their pain [5].

Low back pain is one of the most common complaints among the elderly population and is experienced by 80% of the population at least once in their lifetime. It has adverse effects on the quality of life. If the pain persists for more than 12 weeks, it is known as chronic pain. It can be due to impaired physical functioning, clinical anxiety, depression, lumbar stenosis, spine fracture, inflammatory disease, or nerve root compression [6–8].

The elderly population has been cared for through the proper healthcare system of the country. These issues place a strain on the healthcare system as well as the economy of the country. The elderly’s utilization of healthcare services is influenced by various factors, including socioeconomic, cultural, financial, and geographical resource availability [9].

Several scales are used to assess pain intensity which include: Numerical Pain Rating Scale, Visual Analogue Scale, Verbal rating scales, Face Rating Scale, [10] McGill Pain Questionnaire [11] and Oswestry disability index [12]. Each of these tools is a credible measure of pain and has test-retest reliability. Both the NPRS and the VAS scale are one-dimensional instruments that solely assess pain severity. However, MPQ is a multidimensional questionnaire that includes information regarding affective, sensory and evaluative aspects of pain perception. In both clinical and academic settings, it is a commonly used measurement instrument [5]. These factors led us to decide to translate the SF-MPQ-2 questionnaire into Urdu.

Each of these tools is a credible measure of pain and has undergone test-retest reliability. The Oswestry Disability Index (ODI) is one of them and is a highly reliable tool for assessing disability and pain in people suffering from low back and lumbar radiculopathy. It is one of the most highly regarded measures for determining the quality of life and level of disability in patients with lumbar radiculopathy [12].

Both The NPRS and the VAS scale are one-dimensional instruments that solely assess pain severity. However, The McGill Pain Questionnaire (MPQ) is a multidimensional questionnaire that includes information regarding affective, sensory and evaluative aspects of pain perception. The McGill Pain Questionnaire MPQ was created in 1975 and has quickly become one of the most extensively used questionnaires globally. However, it was the questionnaire is long and is one of the lengthy and time-consuming for patients to complete questions [3]. In 1987, the short form-MPQ (SF-MPQ-2) was designed. This version It takes 2–5 min to administer and this version. This updated version, named Short-Form McGill Pain Questionnaire Version 2 (SF-MPQ-2) or revised version, and was proven to have has excellent validity and reliability [13]. In both clinical and academic settings, it the SF-MPQ-2 is a commonly used measurement instrument [5]. These factors led us to decide to translate the SF-MPQ-2 questionnaire into Urdu, our mother tongue.

Urdu is the most widely spoken language in Pakistan. It is the language that medical professionals use when educating patients and their families about their medical issues. As a result, it is critical to translate the English version of the reliable and valid SF-MPQ-2 questionnaire into Urdu for use among people who speak Urdu as their first language living all across the world. Therefore, the purpose of this study was to translate and validate the English version of the SF-MPQ-2 into Urdu (SF-MPQ-2-U) so that it can be used in research and clinical settings to assess pain.

## Methods

### Instrumentation/measurment tools

#### Short form mcgill pain questionnaire-2

The 22 components of SF-MPQ-2 are used to measure several types of pain, including affective, neuropathic, intermittent, and continuous pain [14]. There are six items in the continuous domain (i.e., “throbbing pain, cramping pain, gnawing pain, aching pain, heavy pain, and tender”), neuropathic domain (i.e., “shooting pain, stabbing pain, sharp pain, splitting pain, electric-shock pain, and piercing”) and intermittent domain (i.e., “hot-burning pain, cold-freezing pain, pain caused by light touch, itching, tingling, or “pins and needles” and numbness”). There are four items in the affective descriptor: tiring-exhausting, sickening, fearful, and punishing-cruel [15]. This questionnaire has been previously translated into different languages, including Persian, Chinese, Japanese, & Turkish [14, 16–18] .

#### Oswestry disability index

The ODI is a self-reported outcome measure that assesses low back pain [12]. There are ten items total, and they are used to determine the level of pain, self-management, lifting, walking, sitting, standing, sleeping, sex life, social life, and travel. Items are given a score between 0 and 5. It has been demonstrated that ODI is a reliable indicator of isokinetic performance, isometric endurance, and discomfort while sitting or standing [19].

### Translation procedure

#### Stage-1: translation process

Two local translators translated the English version into the Urdu language. Each of the translators produced an independent Urdu translation (T1 and T2). Another version, V3, produced by the translators and the principal investigator, removed any ambiguities and chose the most appropriate translations from T1 and T2.

#### Step 2: backward translation

Two translators who were unaware of the English version carried out the backward translation of the V3. They translated the Urdu version (V3) into the Source language (BT1). The purpose of this phase was to make sure that the target language gives the same content that is used in the source language.

After completing the backward translation (BT1), the local translator compared the backward translated English version and the original English version SF-MPQ to rule out any misunderstandings. After analyzing BT1 and Source language, they removed any ambiguities and chose the most appropriate translation.

#### Stage-3: patient testing

To check the understandability of the pre-final version, a pilot study was done on 15 male and female patients (aged 30–60 years) with back pain who were native speakers of the target language.

All the participants with structural abnormalities, any disability, language and memory problems, and who could not read and understand the Urdu language were excluded from the study. The pre-final version was distributed among them, and they were interviewed, patients were asked if they felt there was any difficulty reading or understanding any item. After the mutual discussion of both translator and backward translator, this version was then finalized and chosen to be distributed among the participants as the final translated version.

#### Stage-4: proofreading

An expert committee consisting of medical professionals, Ph.D. Scholars and translators checked for typing mistakes, grammatical errors, and spelling mistakes. No typing, spelling mistakes, or grammatical errors were found in V3. Therefore, the expert committee finalized the translated version 3 (V-3) and approved its distribution to test psychometric properties.

### Psychometric testing

#### Participants

The non-probability convenient sampling strategy was used to select 110 patients. The sample size was calculated according to the guidelines provided by Nicolaou et al., the sample size should be bigger than the number of variables; N > p, which is true in our instance. Gorsuch et al. suggested using 5 cases per indicator. Although any of these ratios—5:1, 10:1, and 20:1—are suitable, 10:1 is the most frequently used. Keeping in mind the methodology of our study, 5:1 was the most suitable option for our study [20]. Total items in our questionnaire: 22 That was multiply by 5 (5*22 = 110). The samples were taken from different physiotherapy clinics, OPDs, and hospitals. Population with chronic low back pain, aged between 30 and 60 years, males and females who could speak and understand the Urdu language and complete the questionnaire without any help, were willing to participate were included in the study according to the inclusion criteria. People who could not understand the Urdu language, had any anatomical, systematic, neurological, or pathological problems other than low back pain, or had any cognitive impairment were excluded from the study. Informed consent was taken from every member who participated in the study.

#### Procedures

The SF-MPQ-2-U and ODI-U and VAS were provided to the patients. A standardized self-administration procedure was followed. Instructions were given verbally to each patient. The translation process started after receiving the ethical approval from the institutional review board. The data collection and patient consent were under the guidelines of Declaration of Helsinki. The translation procedure was governed by the protocol defined by MAPI e-trust services, the original owners of the SF-MPQ-2.

### Data analysis

SPSS version 25 was used to evaluate the data collected from the participants. SF-MPQ-2-U ODI-U and VAS descriptive statistics were analyzed using means, standard deviations, and frequencies. The normality of data was assessed by using the Shapiro-Wilk test.

Convergent Validity, Pearson’s Correlations, ICC and Test-Retest Reliability:

The relationship between SF-MPQ-2-U, ODI-U, and VAS was evaluated for convergent validity. The convergent validity of SF-MPQ-2-U was examined by investigating the correlation between inter-scales. The strength of the correlation between the two domains was determined using Pearson’s correlation coefficient (r) The SF-dependability was evaluated using internal consistency and test re-test reliability. The internal consistency of SF-MPQ-2-U for the total scale was assessed using Cronbach’s alpha and McDonald’s Omega. The intraclass correlation coefficient (ICC), which ranges from 0 to 1, was used to determine test-retest reliability. In order to determine the test-retest reliability, data were collected twice with a gap of 48 h. No intervention was applied to the participants during these 48 h. Internal consistency was checked using Cronbach’s alpha Low ICC numbers (0.5) indicate poor reliability, moderate ICC values (0.5–0.75) show moderate reliability, good ICC values (0.75–0.9) indicate good reliability, and any value above 0.9 indicates great reliability [21].

#### Confirmatory factor analysis

Confirmatory factor analysis of the data was performed using AMOS software, with the goodness of fit index, normed compliance index, and mean square root of estimated errors as the evaluation measures. Maximum likelihood estimation method was used in this analysis. Model fit of the confirmatory factor analysis (CFA) was assessed using the following fit indices: Goodness of Fit index (GFI; acceptable values > 0.90), Normed Compliance Index (NFI; acceptable values > 0.90), Root Means Square Error of Approximation (RMSEA; very good < 0.05, good = 0.05–0.08, mediocre = 0.08–0.10, unacceptable = 0.10 or above [23]), Comparative Fit Index (CFI; acceptable values > 0.90), and Tucker-Lewis Index (TLI; acceptable values > 0.90) [22]. Values more than 0.90 were considered acceptable and indicated a good-fitting model of CFA. RMSEA considered as very good if it is < 0.05, 0.05–0.08 considered as good, 0.08–0.10 considered as mediocre and 0.10 considered as unacceptable [23]. To evaluate the goodness of fit of our proposed model, we used several fit indices, including the AGFI, NFI, and ECVI. The AGFI (Adjusted Goodness of Fit Index) and NFI (Normed Fit Index) are measures of the proportion of variance and covariance accounted for by the model. The ECVI (Expected Cross-Validation Index) evaluates the model’s predictive accuracy[24].

## Results

Out of 110 participants, forty-three were males (39.1%), and 67 were females (60.9%). The mean age of participants was 45.16 years, with a standard deviation of ± 7.58. Out of the total sample size selected for the study, 33(30%) participants were between the age of 31–40 years, 48 (43.6%) participants were between 41 and 50, and 29 (26.4%) participants were between 51 and 60 years. The participants of the study were also categorized according to their socioeconomic background. Out of 110 total participants, two (1.8%) were from the lower class, 85 (77.3%) were from the middle class, and 23(20.9%) were from the upper class. (Table 1)

**Table 1:** Descriptive analysis of sample

Variables	Frequency	Percentage (%)
Gender		
Male	43	39.1
Female	67	60.9
Age (Years)		
31–40	33	30.0
41–50	48	43.6
51–60	29	26.4
Socio-Economics Status		
Lower	2	1.8
Middle	85	77.3
Upper	23	20.9
Weight (Kg)		
51–60	44	40.0
61–70	35	31.8
71–80	16	14.5
81–90	9	8.2
90–100	6	5.5

### Reliability

#### Internal consistency and test-retest reliability

Cronbach’s alpha showed acceptable and statistically significant (p < 0.01) internal consistency of SF-MPQ-2-U of baseline and retest data throughout its four domains (Table-2). Cronbach’s alpha coefficient showed good internal consistency at the first and second visits. McDonalds Omega score for the SF-MPQ-U were 0.918.

**Table 2:** Descriptive statistic, internal consistency and test re-test reliability of SF-MPQ-2-U

Subscale	Items	Base line Mean ± SD	Re-Test Mean ± SD	Cronbach’s Alpha Baseline	Cronbach’s Alpha Re-Test	ICC	95% CI
Continuous	6	4.77 ± 1.53	4.98 ± 1.42	0.799	0.784	0.811	0.755-0.859
Intermittent	6	4.14 ± 1.60	4.30 ± 1.59	0.741	0.730	0.878	0.842-0.909
Neuropathic	6	3.29 ± 1.26	3.47 ± 1.26	0.774	0.744	0.799	0.738-0.850
Affective	4	4.26 ± 1.86	4.40 ± 1.77	0.735	0.795	0.875	0.837-0.908

Abbreviation: SD=Standard deviation. ICC= Intraclass coefficient of correlation. CI=Confidence interval

Intraclass correlation coefficients at 95% confidence interval (p = 0.05) for the Continuous, Intermittent, Neuropathic, and Affective domains were 0.811 (CI = 0.755–0.859), 0.878 (CI = 0.842–0.909), 0.799 (CI = 0.738–0.850), and 0.875 (CI = 0.837–0.908), respectively, which is highly correlated and indicating good reliability (Table 2).

## Discussion

Before starting data collection, permission was obtained from the original authors of SF-MPQ-2, and then the translation procedure began following the ‘authors’ guidelines. The SF-MPQ-2-U was also tested for validity and reliability. The reliability and validity of SF-MPQ-2- has been tested by different authors in different translations [18]. Clinical trials have made use of self-reported scales and methods for evaluating physical pain in adults. Physical pain is regularly assessed in clinical settings to identify what kind of treatment is most likely to have positive impact on a patient’s experience of pain [4]. According to research by Theresa A. et al. limited English proficiency and patient language should be taken into account when assessing pain in the setting of mental health [25]. When patients can understand the question better, they are better able to describe their symptoms. Therefore, it is necessary to give patients a self-reported questionnaire in their language. In Pakistan, this study is the first to translate the English version of the SF-MPQ-2 into Urdu, to the best of our knowledge. Due to linguistic barriers, most patients who visit clinics complaining of pain cannot express their symptoms. Patients report difficulties understanding and adequately responding to the SF-MPQ-2 English version. To rule out this issue, we have translated the English version for our native speakers, whose first language is Urdu. This Urdu version questionnaire can be used in clinical settings, and doctors can offer the finest care when patients are able to accurately describe their problems.

### Participants

The study included 110 participants (43 men, 67 women) with persistent low back pain, aged 30 to 60, who could speak and understand Urdu. The average age was 45.16 years, similar to previous studies. The SF-MPQ-2 was translated using a sample size of 194, and previous studies also had similar sample sizes and demographics. This information highlights the comparability of our study to previous research. (Table 1) [16, 18, 26]. [17, 27]. [18] [17, 27]….

The data was collected from a private physical therapy institution. The participants in our study were middle-aged male and female ranging from the age between 30 and 60 who had persistent low back pain and could speak and understand Urdu. Previous studies included male and female participants with a mean age of 63.7, 16 years or older, more than 18 years old, a history of visceral pain lasting more than three weeks, sub-acute or chronic pain, Neuropathic and non-neuropathic pain, can read and write, and are willing to volunteer for the study sample. People who could not read or write Urdu and those with structural, systemic, or neurological issues were excluded from the study. In similar way, intellectual problems, dementia, psychosis, refusal to engage, and inability to speak that language were all exclusion factors in earlier studies [14, 16, 17].

### Reliability

In our study, the time between test re-tests was set at 48 h to minimize any changes in the patient’s circumstances and ensure that the participants did not recall their answers during the original assessment immediately. This was done to eliminate the possibility of bias [18]. This was comparable to prior research that employed a shorter period of 3 days between test and re-test, and another study collected data once in the morning and once in the afternoon [18] [16, 26].

Cronbach’s alpha demonstrated adequate and statistically significant (p < 0.01) internal consistency. Cronbach’s alpha for previous studies ranged from 0.8 to 0.9 [16, 18]. During the current study Cronbach’s alpha ranged from 0.73 to 0.79 for each subscale, demonstrating acceptable test-retest reliability. When discussed individually the internal consistency for the continuous, intermittent, Affective and Neuropathic domains of SF-MPQ-2-U were 0.79, 0.74, 0.77, 0.79 respectively. The continuous domain score was constant with Thai version (0.79), Persian version (0.75), and acute low back version (0.77) [14, 28, 29]. The internal consistency of the intermittent domain of SF-MPQ-2-U is slightly less than the previous studies including the Thai and Persian translated version of the SP-MPQ-2. However, when internal consistency of the versions with low back pain patients were reviewed a generalized trend of lower internal consistency was found for the intermittent domain as compared to other domains of their respective versions [18, 28]. The internal consistency of the Affective domain of current study was comparable with that of the Sinhala version(0.79) and the Persian version (0.73) [14, 27] finally the neuropathic domain internal consistency for the current study was comparable to that of the Persian version of SF-MPQ-2 [14]. In general, the overall internal consistency of the Urdu version of SF-MPQ-2 scale was satisfactory. The reason of slightly lower scores may suggest the variation due to the specific population considered for this study which included participants with low back pain, in addition to this the cultural perception to pain and its effect on overall life are also a factor in the variation present between current study and studies from other cultures.

Intraclass correlation coefficients with a 95% confidence interval demonstrated acceptable reliability. In case of these results of ICC demonstrated that the SF-MPQ-2-U has an acceptable internal consistency and test-retest reliability. ICCs in prior investigations were 0.973 and 0.027, showing strong reliability. The previous literature showed that the translated versions of SF-MPQ-2 after translation remain valid and reliable compared to the original questionnaire [16, 18].

### Validity

One method used to test SF-MPQ-2-U validity was by assessing its convergent validity. In this study, the ODI-U and VAS scales and the SF-MPQ-2-U were utilized to assess convergent validity. VAS, ODI, SF-36, and WOMAC questionnaires have already been utilized in the literature for convergent validity for the translated SF-MPQ-2 versions [18, 26, 27] .

Pearson’s correlation between scales was r = 0.253, with a significance level of p < 0.01. Previously, the SF-MPQ-2 was significantly correlated with other scales used to evaluate convergent validity [17]. The Pearson’s coefficient r was also considered as the effect size of these correlations. This method of effect size has been mentioned by Rosenthal, R. in their book[30].

The strong positive correlation (p = 0.01) between the SF-MPQ-2-U domains and the ODI-U and VAS components was already presented in the results. However, it is important to note that the SF-MPQ-2-U and ODI-U subscales, except for the intermittent domain of the SF-MPQ-2-U with Walking and Traveling, were positively correlated. The Affective domain of the SF-MPQ-2-U showed moderate correlation values ranging from 0.25 to 0.28 with standing, sleeping, and social life, and it was negatively related to social life (p < 0.01). These findings are in line with previous research that has shown similar relationships between pain measures and disability scales (Table 5).

### Confirmatory factor analysis

Our study of CFA findings revealed a good fitting model, which is similar to another research that found an adequate CFA model of fit whose RMSEA value was 0.0517 and GFI was 0.916 [27] this study showed the CFA results as RMSEA = 0.0517, GFI = 0.916, AGFI0.895. The CFA model showed a four-factor model that fit the data well, which is consistent with the studies of Bupha et al.[28] and Dworkin et al.[15, 29]. The results from our study were also comparable to the results of the original version. All the previous studies conducted for translated SF-MPQ-2 used the Goodness of Fit Index (GFI; >0.90) and the root mean square error of approximation (RMSEA; 0.10) to assess the acceptability of fit of the factor solutions for the CFA[15, 18, 27].

In this study, we looked at the psychometric characteristics of SF-MOQ-2-U Our findings point to the scale’s strong construct validity and internal consistency, which support its potential utility as a clinical tool. Our findings also point out the usefulness of several sub-scales of the measure, such as those linked to pain and its affects in identifying particular symptom clusters that may be helpful for diagnosis and treatment planning. Also, according to our findings, the scale might be helpful for assessing treatment outcomes and tracking treatment progress, particularly in the context of pain management therapies.

### Limitations and Future Research prospects

Future studies are needed to examine not only the psychometric but also the clinometric properties of the McGill Pain Questionnaire since this is an assessment instrument to be used in clinical research and practice with patients. More studies with a variety of conditions should be undertaken in the future. We only focused on one condition and adapted the study to fit that condition and those people. The last stage should be a translation that is not limited to a certain situation. Instead, it should be translated for the entire population, regardless of condition, as was the case with the initial version of SF-MPQ-2. The small number of willing volunteers in the study was a restriction. To achieve better results, this can be done with larger sample sizes. Based on the limitations and gaps identified in our study, we recommend the following specific areas of focus for future research [1] multi-group invariance testing: We recommend performing multi-group invariance testing to determine how well the scale operates across different demographic groups. This will allow us to see if there are any disparities in how individuals from different groups respond to the scale items, and whether the scale can be used to create meaningful group comparisons [2]. Longitudinal invariance testing: We recommend performing longitudinal invariance testing to assess the stability of the scale’s psychometric features across time. This will allow us to establish whether the scale retains its validity and reliability over time, and whether any changes to the scale are required to ensure its utility in longitudinal investigations [3]. Minimum clinically important difference (MCID) estimation: We recommend evaluating MCID values to find the smallest change in score on the scale that is clinically relevant. This will help clinicians and researchers understand the clinical implications of changes in scale scores and will aid in establishing the scale’s utility in clinical settings [4]. Rasch analysis: We recommend performing Rasch analysis to see whether the scale items function as expected based on the Rasch model. This will allow us to establish whether the items are measuring the same underlying concept, whether the items are arranged correctly in terms of difficulty, and whether there are any biases in how individuals respond to the things.

## Conclusion

In conclusion, the SF-MPQ-2-U is considered to have sufficient validity and reliability for measuring different types of pain in patients with low back pain who speak Urdu. In order to make the questionnaire more valid and reliable, it is recommended for researchers to do in-depth research on a larger sample size.

### Validity

#### Convergent validity at inter-scale level

There was statistically significant positive and strong correlation between Continuous and Intermittent variables (r = 0.719), Continuous and Neuropathic variables (r = 0.635), Continuous and Affective (r = 0.639), Intermittent and Neuropathic variables (r = 0.717), intermittent and affective variables (r = 0.746, p < 0.01) and between Neuropathic and Affective variables (r = 0.655). The effect size of these correlations was good at (r = 0.719, 0.635, 0.717, 0.655) respectively (Table 3).

The Correlation of inter-scale after retest followed the same trend giving a statistically significant correlation between continuous and intermittent, Continuous and Neuropathic, Continuous and Affective, Intermittent and Neuropathic, Intermittent and Affective, Neuropathic and Affective with 0.693, 0.592, 0.600, 0.663, 0.708, and 0.607 respectively which showed statistically significant and positive Correlation. (Table 3)

**Table 3:** Correlation between inter-scales (SF-MPQ-2-U) of baseline &test-re-test data

	Continuous Baseline	Intermittent Baseline	Neuropathic Baseline	Continuous Re-test	Intermittent Re-test	Neuropathic Re-test
Intermittent	0.719	-		0.693		
Neuropathic	0.635	0.717	-	0.592	0.663	-
Affective	0.639	0.746	0.655	0.600	0.708	0.607

#### Convergent validity between two scales

The correlation between SF-MPQ-2-U and ODI-U at the baseline was 0.547. The baseline correlation between Short Form McGill pain questionnaire 2-U and VAS, where VAS is a pain assessment scale, was 0.558, while the baseline correlation between ODI-U and VAS was 0.596.

The Pearson’s correlation between scales was r = 0.253, which is statistically significant. (Table 4)

**Table 4:** Convergent validity, correlation between SF-MPQ-2-U, ODI-U And VAS of baseline & test-retest data

Variables Baseline	SF-MPQ-2-U Baseline	ODI-U Baseline	Variables Retest	SF-MPQ-2-U Test-rest	ODI-U Test-rest	VAS Test-rest
ODI-U	0.547^**^	-	ODI-U	0.561^**^	-	
VAS	0.558^**^	0.596^**^	VAS	0.463^**^	0.640^**^	-

Abbreviation: ODI = Oswestry disability index-Urdu, VAS = Visual analogue scale.

The correlation between SF-MPQ- 2-U and ODI-U after the re-test was 0.561. The correlation between Short Form McGill pain questionnaire 2-U and VAS after the re-test was 0.463, and the correlation between ODI-U and VAS after the re-test was 0.640. The SF-MPQ-2, ODI-U, and VAS all had a statistically significant and positive correlation. (Table 4)

The correlation between the SF-MPQ-2-U domains and the ODI-U and VAS elements was calculated. Table 5 shows a significant and positive association between the SF-MPQ-2-U domains and most ODI-U features. However, there is negative correlation between the social life element of the ODI scale and the SF-MPQ-2-U domains. A significant positive correlation exists between the domains of the SF-MPQ-2-U and the total results of ODI-U and VAS. All of these scales revealed a statistically significant positive and strong correlation. (Table 5)

**Table 5:** Correlation between subscales of SF-MPQ-2-U, ODI-U and VAS

Convergent Validity	Continuous	Intermittent	Neuropathic	Affective	SFMPQ-2-U	VAS
ODI	0.508**	0.484**	0.411**	0.506**	0.547**	0.596**
Pain intensity	0.411^**^	0.373^**^	0.334^**^	0.431^**^	0.442^**^	0.724^**^
Personal care	0.351^**^	0.368^**^	0.286^**^	0.405^**^	0.403^**^	0.457^**^
Lifting	0.462^**^	0.428^**^	0.285^**^	0.444^**^	0.467^**^	0.494^**^
Walking	0.230^*^	0.245^*^	0.252^**^	0.333^**^	0.299^**^	0.312^**^
Sitting	0.404^**^	0.446^**^	0.395^**^	0.513^**^	0.505^**^	0.348^**^
Standing	0.356**	0.377**	0.333**	0.250**	0.381**	0.473**
Sleeping	0.400**	0.370**	0.360**	0.285**	0.408**	0.222*
Social life	-0.001	-0.038	0.1	0.229*	0.077	0.129
Travelling	0.318**	0.207*	0.136	0.141	0.237*	0.294**
Employment	0.436**	0.426**	0.278**	0.448**	0.457**	0.490**
VAS	0.596**	0.508**	0.523**	0.410**	0.499**	1.00

#### Confirmatory factor analysis

Table-6 shows that the GFI and NFI for SF-MPQ-2-U were 0.952 and 0.965, respectively, were used to assess the fit of the CFA factor solution. The RMSEA was 0.082 that was acceptable fit. The Comparative Fit Index and Tucker-Lewis Index have also been calculated, whose values are CFI = 0.92 and TLI = 0.94, which are considered an acceptable fit [22]. (Table 6)

**Fig. 1:**
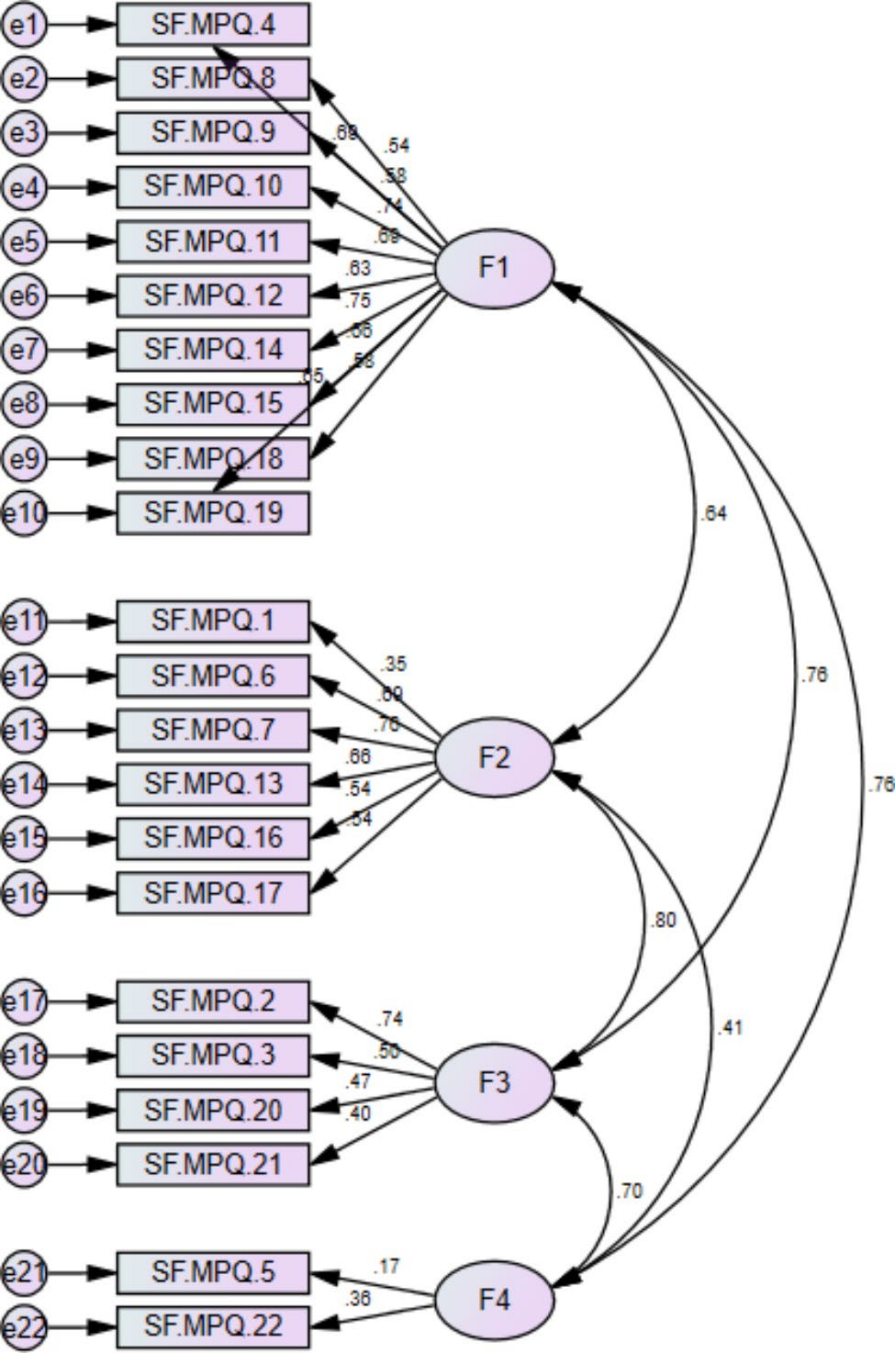
Confirmatory analysis diagram. GFI = 0.805, AGFI = 0.757, NFI = 0.686, NFI = 0.868, TLI = 0.838, CFI = 0.858, RMSEA = 0.070, ECVI = 3.769

Based on the results of the confirmatory factor analysis (CFA), the goodness of fit indices indicate that the model fits the data moderately well. The goodness of fit index (GFI) was 0.805, indicating that 80.5% of the variance in the observed variables was explained by the model. The adjusted goodness of fit index (AGFI) was 0.757, indicating that the model fit the data reasonably well. The normed fit index (NFI) was 0.686, indicating a moderate fit between the model and the data. The comparative fit index (CFI) was 0.858, indicating a good fit between the model and the data. The Tucker-Lewis index (TLI) was 0.838, indicating a moderate fit between the model and the data. The root mean square error of approximation (RMSEA) was 0.070, indicating a reasonable fit between the model and the data. Finally, the expected cross-validation index (ECVI) was 3.769, which is a measure of the model’s predictive accuracy. Overall, the results suggest that the CFA model has a moderately good fit to the data, but there may be some room for improvement. (Figure 1)

**Table 6:** Confirmatory factor analysis of SF-MPQ-2-U

SF-MPQ-2-U	GFI	NFI	RMSEA	CFI	TLI
Total	0.805	0.686	0.070	0.858	0.838

## Data Availability

The data set used for the statistical analysis and interpretation during the current study is available from the corresponding author at a reasonable request.

## Abbreviations

SD: Standard deviation
ICC: Intraclass coefficient of correlation
CI: Confidence interval
